# Studying human culture with small datasets and evolutionary models

**DOI:** 10.1098/rspb.2023.0753

**Published:** 2023-06-28

**Authors:** Alberto J. C. Micheletti, Erhao Ge, Liqiong Zhou, Yuan Chen, Juan Du, Ruth Mace

**Affiliations:** ^1^ Department of Anthropology, University College London, 14 Taviton Street, London, UK; ^2^ State Key Laboratory of Grassland and Agro-Ecosystems, College of Ecology, Lanzhou University, 222 Tianshui South Road, Lanzhou, Gansu Province 730000, People's Republic of China

**Keywords:** celibacy, cultural behaviours, inclusive fitness, small data, anthropological research

In our paper, we aimed [1] to advance and assess the hypothesis that committing a son to lifelong religious celibacy is an adaptive way for parents to reduce competition between their sons and increase their own reproductive success. We formalized this hypothesis with an inclusive fitness model identifying the conditions under which this behaviour is favoured by natural selection and tested whether these conditions—that is, the assumptions of our model—are met in a Tibetan population where parents often sent a son to the monastery until recently. Some of the goals and methods of studies such as ours, which seek to understand the extent to which human cultural behaviours are adaptive using data from the field and evolutionary models, are sometimes misunderstood, leading to confusion [2]. We take the opportunity offered by the comment by von Pein *et al*. [3] to once again clarify this confusion, while confirming our results.

As we explain in the introduction of our paper, the extent to which the evolution of human cultural behaviours is shaped by the inclusive fitness interests of their bearers has been the subject of an intense debate in the evolutionary human sciences since their inception in the 1970s and 1980s [4]. To be clear, this is not a trivial issue, but instead amounts to one of the greatest challenges in the study of cultural evolution. The debate is far from being resolved. In recent years, much empirical and modelling work has aimed to bring clarity to this question (e.g. [5,6]). In our work [1], we did so by assessing the extent to which lifelong religious celibacy is adaptive using sociodemographic data we collected in a Tibetan Buddhist population in western China and an inclusive fitness model.

Progressively addressing this question has sometimes stalled because of the unhelpful dichotomy between adaptation and culture assumed by many scholars (including von Pein *et al*. [3]). Micheletti *et al.* [2] have argued that this dichotomy stems from a confusing use of the term cultural evolution to identify both a phenomenon—cultural change in time—and a theory for it—the role that transmission biases may play in this change. Once this confusion is clarified, it becomes clear that adaptation to local ecology and cultural transmission are not alternative explanations [2,7]. Many human behaviours are culturally transmitted, and yet they can be shaped, at least to a degree, by the inclusive fitness interests of their bearers, if individuals can decide whether to adopt or reject them [2,8,9] and they do so in a way that is sensitive to payoffs (as it has been shown to often be the case [10,11]).

Given these considerations, a kin selection model assuming genetic transmission is a valid tool to assess the inclusive fitness costs and benefits of a behaviour and the conditions under which it can be adaptive. As we discussed at length in section 3 of our paper [1], it is a heuristic model, rather than a mechanistic one. We are not interested here in modelling how celibate behaviour is transmitted, but in understanding to what extent it is shaped by inclusive fitness interests. Our inclusive fitness model of male lifelong celibacy showed that this practice can be favoured by selection—that is, can be adaptive—and reach non-negligible levels if we assume that, first, the decision to commit to celibacy is under parental control and, second, monks make their lay brothers more competitive, increasing their reproductive success. A model is a mathematical formalization of a hypothesis that allows us to ascertain the logical consequences of a set of assumptions, generating an explanation and predictions [12]. Therefore, we tested whether our assumptions are realistic, using sociodemographic data we collected in Tibetan Buddhist villages in western China. The statistical analyses of reproductive success we performed allowed us to understand if the conditions leading to selection favouring celibacy are met in this population. If they are, this would suggest that our model has some explanatory power.

The first assumption of our model is that the decision to commit to lifelong celibacy is taken by an individual's parents, not by the individual himself. This condition is met in our population. Until recently, it was common for parents in the area we study to send one of their sons to the local monastery to become a celibate Buddhist monk when only 7–10 years of age [1,13], so the decision is made by the parents or is heavily influenced by them. The second assumption is that monks make their lay brothers more competitive, that is more likely to secure one of a limited number of ‘reproductive spots’, that is more chances to reproduce, generating a set number of offspring. Competition for reproductive spots is very often assumed in social evolution theory to make evolutionary models in group-structured populations mathematically tractable and broadly captures the notion that resources are limited at a local scale [14,15]. In our case, men will vary in the number of children they have, so the best way to test the validity of the assumption that monks make their brothers more competitive is to test if men with a monk brother have more children than those who do not.

In the paper, we first considered all living men born between 1961 and 2000 in our sample. Note that, in these cohorts, the average number of children for men who are not monks is 1.53 (s.d. = 1.22, *n* = 934) and for women is 1.64 (s.d. = 1.17, *n* = 929)—a small number of people did not have an identifiable father (so women do not have lower reproductive success, *contra* von Pein *et al*. [3]). We found that those who have a monk brother have 1.75 times more children than men whose brothers are not monks and a similar number of children to men who are only sons [1]. As, in our sample, first-born sons are more likely to inherit and less likely to become monks [13], we then restricted this analysis to men who are first-born children (we have clarified this with a wording correction) and found that the effect remained strongly significant [1]. Here, we perform a third analysis, further restricting our sample to men who are first-born children and have only one younger brother so as to control for any effects of number of brothers. We find that men with a monk brother have 1.59 times more children than men whose brother is not a monk (multilevel Poisson regression, *b* = 0.466, 95% CI: [0.113, 0.820], *n* = 134, *p* = 0.01; table 1). Note that, among men who are not monks and have a brother, younger brothers tend to have fewer offspring on average than firstborn children (0.45 (s.d. = 0.99) versus 1.08 (s.d. = 1.20)); considering that monks are generally later-born children [13], the increase in reproductive success for men with a monk brother need not be twofold for the practice to be adaptive (*contra* [3]). Overall, this additional analysis confirms that our model assumption that men with a monk brother make their lay brothers more competitive appears to be met in our population, even despite the fertility restrictions that have been in place since the late 1980s.

**Table 1.** Reproductive success of men who are first-born children with only one younger brother. Parameter details for the full model of determinants of the number of living children for 134 men (63 men are monk brothers and 71 are not monk brothers). The key variable is in bold.

variables	estimate	95% CI	*Z*-value	*p*-value
(intercept)	1.395	(−0.413, 3.202)	1.512	0.13
birth year cohort				
ref: 1961–1970				
1971–1980	0.108	(−0.295, 0.511)	0.524	0.6
1981–1990	0.385	(−0.032, 0.802)	1.811	0.07
1991–2000	−1.418	(−2.045, −0.791)	−4.431	<0.001
wealth	−0.073	(−0.239, 0.094)	−0.856	0.392
distance to town	−0.024	(−0.059, 0.010)	−1.375	0.169
ref: 0 sisters				
≥1 sisters	0.134	(−0.366, 0.634)	0.524	0.6
**ref: not monk brother**				
** **monk brother****	**0.466**	**(0.113, 0.820)**	**2.586**	**0.01**
random factor				
village (variance)	<0.001			

We then explored how this benefit is accrued. In this socially monogamous population with a recent past of polygyny and polyandry, men with a monk brother could have greater reproductive success because they have more mating partners, because their wives are more fertile or both. We opted to assess the age at first birth of women who have a monk brother-in-law. Such analyses are common in the anthropology literature; nonetheless, here we clarify the reasons for our choice. Women have been limited to three births since the late 1980s, so we investigated age at first birth—a trait that could have led to higher reproductive success were fertility not artificially constrained and also gives fitness advantages even if fertility is constrained, as it shortens generation times [16,17]*.* Our age-at-first-birth analysis shows that women with a monk brother-in-law have their first child earlier (see section 2c, fig. 1*d* and electronic supplementary material, tables S11 and S12 for [1]; we issued a correction to add Kaplan–Meier estimates for mean age at first birth in the legend of electronic supplementary material, table S11). While von Pein *et al.* [3] argue that our tests of reproductive success are ‘redundant’ (despite asking for further analysis), we have performed an additional multilevel Poisson regression focusing on number of living children: this suggests that women who have a monk brother-in-law have 1.24 times more children than women who do not (*b* = 0.214, 95% CI: [0.068, 0.361], *n* = 929, *p* = 0.004; table 2) as we found for men. So brothers of monks are made somehow more attractive—and can thus secure wives earlier—or are able to provide their wives with more material resources. In either case, these results suggest that they would have accrued fitness benefits mostly through their wife, rather than polygamous relationships.

**Table 2.** Reproductive success of monk sisters-in-law. Parameter details for the full model of determinants of the number of living children of 929 women (91 have a monk brother-in-law and 838 do not). The key variable is in bold.

variables	estimate	95% CI	*Z*-value	*p-*value
(intercept)	0.855	(0.359, 1.351)	3.376	0.001
birth year cohort				
ref: 1961–1970				
1971–1980	0.17	(0.032, 0.307)	2.412	0.016
1981–1990	0.204	(0.071, 0.337)	3.005	0.003
1991–2000	−0.622	(−0.819, −0.426)	−6.203	<0.001
wealth	−0.018	(−0.070, 0.034)	−0.688	0.491
distance to town	−0.005	(−0.014, 0.005)	−0.984	0.325
ref: 0 brothers				
≥1 brothers	−0.643	(−0.836, −0.449)	−6.507	<0.001
ref: 0 sisters				
≥1 sisters	−0.644	(−0.888, −0.401)	−5.184	<0.001
**ref: not sister-in-law of monks**				
** sister-in-law of monks**	**0.214**	**(0.068, 0.361)**	**2.872**	**0.004**
random factor				
village (variance)	<0.001			

Finally, we assessed whether men with a monk son have higher reproductive success than men without one. For these father analyses, we needed three generations, so we used living and deceased men in birth year cohorts less than or equal to 1950–1980 (as we described in the main text and the electronic supplementary material of [1]). We performed two father analyses: the first comprised all men with at least one child and the second only men with at least one son (we have eliminated any ambiguity in terms used to report these analyses in our paper [1] with a correction). Controlling for number of children, men with a monk son have more grandchildren than the rest in both cases, with effect sizes *b* = 0.141 and *b* = 0.148, respectively [1]. Here, we perform a third analysis, further restricting our sample to men with at least two sons (multilevel Poisson regression, *b* = 0.132, 95% CI: [−0.151, 0.415], *n* = 236, *p* = 0.361; table 3)*.* Given that this subsample is much smaller than the previous, we have lower statistical power, which may go some way towards explaining the larger *p*-value. Nonetheless, it is worth noticing that the effect estimate is still comparable to that in the previous analyses. As we said in the paper, ‘Having one fewer potentially reproductive son should result, all else being equal, in a lower number of descendants in the following generation’ [1, p. 3]. We do find it of interest that, independently of how it is measured, having a monk son does not appear to be a cost. This strongly suggests that the fitness costs to the celibate are balanced by fitness gains to other family members.

**Table 3.** Reproductive success of monk fathers, men with at least two sons. Parameter details for the full model of determinants of the number of living grandchildren for 236 men with at least two sons (140 men are monk fathers and 96 are not monk fathers). The key variable is in bold.

variables	estimate	95% CI	*Z-*value	*p-*value
(intercept)	−0.073	(−1.250, 1.104)	−0.121	0.904
birth year cohort				
ref: ≤1950				
1951–1960	0.175	(−0.100, 0.450)	1.246	0.213
1961–1970	−0.143	(−0.468, 0.182)	−0.861	0.389
1971–1980	−1.537	(−1.937, −1.137)	−7.532	<0.001
wealth	−0.014	(−0.131, 0.103)	−0.235	0.814
distance to town	0.011	(−0.010, 0.032)	1.033	0.302
ref: 2 offspring				
3+ offspring	0.125	(−0.136, 0.385)	0.936	0.349
**ref: 0 monk sons**				
** ≥1 monk sons**	**0.132**	**(−0.151, 0.415)**	**0.913**	**0.361**
random factor				
village (variance)	<0.001			

Running this additional analysis gave us the chance to consider whether the absence of increased reproductive success for monk fathers would suggest that our model is incorrect. Actually not inclusive fitness models such as ours allow us to assess in which direction selection is expected to act and identify the frequency of a given behaviour predicted at equilibrium (the ‘convergence-stable strategy’ [14,15]). For this reason, if the system has not reached equilibrium, we would expect an advantage for a monk father. If instead the system has reached equilibrium, we would expect no difference. It is hard to say if the system we studied empirically is at equilibrium. Monasteries were closed in 1958, and parents could not make their sons monks for 20 years or so. But after they reopened in the late 1970s and early 1980s, the practice quickly rebounded. We think that parents simply went back to the strategy that they had usually followed before, so the system could be considered at equilibrium.

As we explain in section 2a of our paper [1], the population has been under fertility-limiting government policies since the 1980s. Therefore, the magnitude of the effects in terms of offspring numbers that we have explored above no longer reflects the conditions in which the behaviour of interest first arose—as we mentioned in the discussion of the original paper [1]. This is a common problem in evolutionary anthropological investigations since the demographic transition: for example, the widespread availability of modern contraception has reversed the correlation between number of sexual partners and number of offspring [18]. These issues favour human behavioural ecologists to seek a wide range of possible measures of reproductive success to seek evidence for their hypotheses, as we have done. Moreover, they focus more on the direction of the effects than their magnitude, as this might have changed due to recent social, economic or technological changes. Our results suggest that the assumptions of our model are realistic for this population and effects are in the expected direction. For these reasons, we stand by our hypothesis that that the practice of sending a son to the monastery has been shaped heavily by the inclusive fitness interests of the monks' families.

Anthropological studies tend to focus on small populations. Generally, as in this case, we have assured ethics committees that our data will not make individuals identifiable, which is why some or all of the variables cannot always be made public. In our case, access to all the variables requested in the comment, including number of offspring, household wealth and distance from the county capital, would make 43% grandfathers uniquely identifiable. In the light of the increased requirements to make data available, studies of protected data (which include variables like religion, wealth, where you live, etc.) from small populations are caught between a rock and a hard place. This issue has not been fully resolved and the field will have to think about it some more. Different kinds of data can sometimes be in separate files that cannot be linked, but—if reproductive success is the focus—one generally needs the whole dataset. It is helpful if communities are not named, but of course anthropologists give a high value to context, and those interviewed obviously know the location if the study only has one location. Panel data of 450 000 individuals clearly allows for anonymity and completely different kinds of studies and statistics.

von Pein *et al*. [3] do not suggest any alternative hypotheses. The replication crisis has encouraged researchers and journals to put time and resources into the continued review of published papers. We hope this does not divert human behavioural scientists from the increasingly challenging task of generating new tests of new or old hypotheses from work in the field, before much of the anthropological diversity around us disappears.

## Data Availability

Data files for the sociodemographic study in the original paper are available from the Dryad Digital Repository: https://doi.org/10.5061/dryad.t76hdr83f [19] (we reduced the number of covariates to protect the identity of the participants). Similarly abridged datasets for the three additional analyses in this reply can be made available.
